# Do nurses suffer from insomnia during the Covid-19 pandemic? a cross-sectional study led in Morocco

**DOI:** 10.1192/j.eurpsy.2023.1681

**Published:** 2023-07-19

**Authors:** I. Hanine, M. Chtibi, S. Belbachir, A. Ouanass

**Affiliations:** 1 Psychiatry, Military Hospital Mohammed V of Rabat, Rabat; 2 Psychiatry, Arrazi university psychiatric hospital, Sale, Morocco

## Abstract

**Introduction:**

Nurses are one of the pillars of the health system, their constant presence with the patients requires a sequence of shifts and nights in the hospital, this aspect has been accentuated during the new pandemic, and undoubtedly impacts their sleep.

**Objectives:**

We propose to study in this paper the effect of on-call duty on the quality of sleep of nurses.

**Methods:**

We used a questionnaire made of two parts, we managed to explore in the first par sociodemographic status of our nurses, the second part was the French version of ISI (Insomnia Severity Index) exploring insomnia, satisfaction of sleep and their functioning.

**Results:**

Regarding descriptive statistiques, from our 90 results, the mean age was 30,9 +/- 6.63, women were equal to men in this study 5% had depressive disorder and 2% anxious disorder, in this study: 68,9% had insomnia 2,5% of them has severe insomnia.

**Conclusions:**

Indeed, insomnia, the satisfaction regarding sleep amongst nurses and there day to day functioning was altered due to recent pandemic.

**Disclosure of Interest:**

None Declared

